# A protective role of SRC-1 against aging associated cognitive decline

**DOI:** 10.18632/aging.206311

**Published:** 2025-08-27

**Authors:** Hesong Liu, Yongjie Yang, Jonathan C. Bean, Yang He, Hailan Liu, Rambabu Majji, Chen Liang, Nan Zhang, Meng Yu, Longlong Tu, Qingzhuo Liu, Yue Deng, Kristine M. Conde, Na Yin, Mengjie Wang, Yongxiang Li, Junying Han, Sanika Vattakuzhiyil Jossy, Megan Elyse Burt, Hari Krishna Yalamanchili, Chunmei Wang

**Affiliations:** 1 USDA/ARS Children's Nutrition Research Center, Department of Pediatrics, Baylor College of Medicine, One Baylor Plaza, Houston, TX 77030, USA; 2 Jan and Dan Duncan Neurological Research Institute, Texas Children's Hospital, Houston, TX 77030, USA

**Keywords:** aging, dementia, SRC-1, S100A6

## Abstract

Introduction: Research indicates a strong correlation between obesity and the risk of dementia, both are linked to steroid receptor coactivator-1 (SRC-1), a transcriptional coactivator.

Methods: We used RNA sequencing analysis (RNA-Seq) to investigate the transcriptome of SRC-1-KO mice, and identified S100 calcium-binding protein A6 (S100A6), an AD associated gene, as one target of SRC-1. We tested cognitive behaviors in SRC-1-KO mice and mice with a humanized SRC-1 mutation (SRC-1^L1376P^), and performed promoter luciferase assays on S100A6.

Results: Loss of SRC-1 caused alterations in gene signatures that are commonly associated with neurodegenerative diseases, including AD, and diminished the neural plasticity of the hippocampal CA1 neurons. Both SRC-1-KO and SRC-1^L1376P^ mice displayed early signs of contextual memory impairment at 6 months of age. Mechanistically, SRC-1 significantly promoted the expression S100A6.

Conclusion: We identified a protective role of SRC1 against aging associated cognitive decline, potentially by promoting the expression of S100A6.

## INTRODUCTION

About 36% of the elderly population (at an age of 70–75) experience mild memory loss, and about 32% are affected by Alzheimer’s disease (AD) [1]. Owing to the aging of populations worldwide, dementia is reaching epidemic proportions, with a large human, social and economic burden. AD is the most common cause of severe memory loss in the elderly. Despite the tremendous efforts in the field of cognitive regulation, the pathophysiology underlying memory decline is not fully understood, and effective treatments are limited.

Research indicates a strong correlation between obesity and an increased risk of dementia and Alzheimer’s disease [2–6]. The brain is rich in various nuclear receptors and transcription factors that play a crucial role in maintaining metabolic equilibrium and cognitive functions. Notably, thyroid hormone receptor [7], glucocorticoid receptor [8], estrogen receptor [9], peroxisome proliferator-activated receptor gamma [10], signal transducer and activator of transcription-3 [11], and Forkhead Box O1 [12], etc. are among those implicated in both metabolic and Alzheimer’s diseases progression. These nuclear receptors and transcription factors depend on coactivators like steroid receptor coactivator-1, 2 and 3 (SRC-1, 2 and 3) [13] for their transcriptional activities. SRC proteins are prevalent in the brain, especially in areas like the hippocampus and hypothalamus [14, 15]. Studies have shown that the deletion of SRC-1 (encoded by *Ncoa1*) gene globally or from the hypothalamus in mice leads to obesity [16, 17]. A particular gene variant, SRC-1^L1376P^ mutation, has been linked to early-onset obesity in children (body mass index standard deviation score (BMI SDS) >3.5; age of onset <10 years), and has been confirmed to induce obesity in mice as well [17, 18]. Despite that loss of SRC-1 does not accelerate the development of AD [19], another study showed that RNAi-mediated knockdown of SRC-1 in the hippocampal CA1 impairs memory [20, 21]. Thus, the dysfunction of SRC-1 is linked to both obesity and memory loss. However, it is unclear whether and how SRC-1 contributes to the aging associated dementia.

Earlier studies have indicated a reduction in SRC-1 expression in the brains of middle-aged individuals [22]. In this study, we confirmed the age associated decline of SRC-1 and further explored the differences in brain gene expression profiles between wild type C57BL/6 (WT) and SRC-1-KO mice. We performed cognitive behavioral tests on WT mice, SRC-1-KO mice and mice with the humanized SRC-1^L1376P^ mutation across various age stages to assess the role of SRC-1 in cognitive functions. Finally, we identified the stimulatory effect of SRC-1 on the expression of S100A6 and S100A11, which are genes potentially associated with AD and memory deficits.

## METHODS

### Mice

Care of all animals and procedures were approved by the Institutional Animal Care and Use Committee of Baylor College of Medicine (AN-5479 and AN-6098). Mice, including SRC-1-KO mice [16] and SRC-1^L1376P^ mutant mice [17], were housed in a temperature-controlled room at 22–24°C using a 12-h light, 12-h dark cycle. All these mice were fed with regular chow (5V5R, PicoLab). Food and water were provided ad-libitum.

### RNA-Seq and analysis

Total hypothalamic RNA was isolated from SRC-1-KO mice and littermates at the age of 20 weeks using the RNeasy Lipid Tissue Mini Kit (Qiagen). Three RNA samples for each group were sent to Genomic and RNA Profiling Core (GARP) at Baylor College of Medicine for sequencing. One hundred-fifty base pair paired end reads were aligned to Genome Reference Consortium Mouse Build 39 reference genome (Mus musculus genome assembly GRCm39 NCBI. https://www.ncbi.nlm.nih.gov/data-hub/assembly/GCF_000001635.27/) using STAR 2.7.9a using option “--outSAMtype BAM SortedByCoordinate” [23]. Output from STAR was then quantified using “featureCounts” function from Subread v2.0.3 using default options [24].

Differential expression was calculated using DEseq2 1.36.0 with test set to “Wald” [25] with R 4.2.2 within RStudio 2022.07.2 Build 576. Differential expression results were filtered for genes that were 1.5-fold either up or down with an adjusted *p*-value <0.05 using dplyr 1.0.10. These genes were used to produce two list of genes that were then used to create molecular function gene ontology utilizing “gost” function from gprofiler2 0.2.1 with options “organism = ‘mmusculus’, ordered_query = FALSE, evcodes = TRUE, correction_method = ‘gSCS’, domain_scope = ‘annotated’, exclude_iea = TRUE, sources = ‘GO:MF’”.

An additional enrichment analysis was conducted using Gene Set Enrichment Analysis (GSEA, version 4.3.2) [26] with the “preranked” option. Genes were first rank ordered using the algorithm log_2_(fold change) X - log_10_ (adjusted *p*-value). Options on GSEA were set to: gene set database “c2.cp.kegg.v2022.1.Hs.symbols.gmt”, chip platform Mouse_Gene_Symbol_Remapping_Human_Orthologs_MsigDB.v2022.1.Hs.chip”, enrichment statistic “classic” and number of permutations “100,000”. Other options were left on default settings. RNA-Seq results were visualized using ggplot2.

Alternative splicing was detected using rMATS turbo (v4.1.24) [27], in Python 2.7.18. Genes splicing events were considered significant if the absolute inclusion level difference was greater than 0.2 and adjusted *p*-value was less than 0.05.

### Secondary analysis of published scRNA-Seq data

Data were obtained from GSE152506 [28]. Raw counts were first normalized using the log_2_ counts per 10,000 formula. Data from either hypothalamus or hippocampus were subset then compared across age using FindMarkers feature of Seurat 5.1.0, Wilcox test followed by Bonferroni correction for multiple comparisons [29]. Data were plotted using VlnPlot feature of Seurat with boxplots superimposed on top.

### Secondary analysis of published bulk RNA-Seq data

Gene expression data for both human and mouse brain tissues were obtained from publicly available sources. Human bulk tissue RNA-seq data were downloaded from the GTEx portal (https://www.gtexportal.org/home/downloads/adult-gtex/bulk_tissue_expression), including brain regions such as the Cerebellum, Cortex, Frontal Cortex, Hippocampus, and Hypothalamus. Corresponding sample metadata, including donor age information, were also downloaded from GTEx and used to stratify samples into two age groups: 20–40 years (combining 20–29 and 30–39 years) and 60–80 years (combining 60–69 and 70–79 years). Differential gene expression (DEG) analysis was performed using DESeq2 [25] on normalized count data. Log-transformed expression values were visualized with violin plots, with age groups on the x-axis and expression on the y-axis. Statistical significance was denoted by asterisks (^*^ for *p* < 0.05, ^**^ for *p* < 0.01). For mouse data, hippocampal gene expression profiles were obtained from the GEO dataset GSE179698, which includes RNA-seq samples from 6- and 18-month-old mice. Expression values for Ncoa1, S100a6, and S100a11 were extracted from the dataset, and log-transformed values were visualized using box-and-whisker plots.

### Electrophysiology

Mice (males, 6 weeks of age) were perfused and brain slices were prepared for electrophysiology recording as we did before. Briefly, pyramidal neurons in CA1, identified based on their location and morphology, were visualized and recorded. Whole-cell recordings were performed on CA1 pyramidal neurons. Evoked EPSCs (eEPSCs) and EPSC based LTP were recorded as we did before [30].

### Immunofluorescence

WT mice (males, 10 weeks of age) were perfused and brains were sectioned and processed for immunohistochemistry staining for SRC-1 and three cell markers. Briefly, brain sections were blocked (3% Normal donkey serum) for 1 h, incubated with Rabbit anti-SRC-1 (#2191, Cell Signaling Technology) on shaker at 4°C for overnight, followed by 3 washes with 1X PBS and then the incubation of the donkey anti-rabbit AlexaFluor 488 (A21206, Invitrogen) for 2 h. After three washes, each series of brain sections were then incubated with one of the cell marker antibodies respectively. Cell marker antibodies include Mouse anti-NeuN (ab104224, Abcam) as neuron maker, Chicken anti-GFAP (ab4674, Abcam) as astrocyte maker and Goat anti-Iba1 (ab5076, Abcam) as microglial cell marker. All the brain sections were then incubated with the donkey anti-mouse/chicken/goat AlexaFluor 594 (A21203/A11042/A11058, Invitrogen) for 2 h. After the last wash and dry, slides were cover-slipped, and images of the hippocampus were captured using a fluorescence microscope.

### Behavioral tests

The Novel Object Recognition (NOR) test was conducted over two consecutive days to assess memory in mice using a modified protocol [31]. On the first day, each mouse was acclimated to the testing room for 30 minutes, followed by a 20-minute habituation period in an empty Open Field (OF) box (40.64 cm × 40.64 cm). Subsequently, two identical objects were placed in opposite corners of the OF box. The mouse was then allowed to explore the arena and the objects freely for 15 minutes with an overhead camera to record its behavior for subsequent analysis. After the 15 minutes session, the mouse was returned to its home cage, and the OF box and objects were thoroughly cleaned with soap and water to eliminate olfactory cues. On the second day, following a 30-minute acclimatization to the testing room, the mouse was then reintroduced to the same OF box. One of the familiar objects was replaced with a novel object that differed in both shape and color. The mouse was again allowed to explore the arena for 15 minutes, during which behavior was recorded. After the session, the mouse was returned to its home cage, and the testing apparatus was cleaned as described above. The Discrimination Index was calculated as the time interacted with the novel object divided by the total time that the mouse interacted with both objects. The water maze test (RAWM) and fear conditioning test were performed as we did previously [30].

### Q-PCR validation of gene expression in the hypothalamus and hippocampus

To examine gene expression, C57BL/6 control and SRC-1-KO mice (male, 4 months of age) were sacrificed, and the hypothalamus and hippocampus were quickly collected. Total RNA was isolated using TRIzol Reagent (Invitrogen) and 2 μg of total RNA was reverse-transcribed to cDNA using a High-Capacity cDNA Reverse Transcription Kits (Invitrogen). Q-PCR was performed on a CFX384 Real-Time System (Bio-Rad) using SsoADV SYBR Green Supermix (Bio-Rad). Primer sequences for S100a6: forward 5′-GAAGGTGACAAGCACACCCT-3′ and reverse: 5′-CCCAGGAAGGCGACATACTC-3′; for S100a11: forward 5′-AAGTACAGCGGGAAGGATGGA-3′ and reverse 5′-ATGCGGTCAAGGACACCAG-3′; for Cyclophilin: forward 5′-TGGAGAGCACCAAGACAGACA-3′ and reverse 5′-TGCCGGAGTCGACAATGAT-3′. The expressions of S100a6 and S100a11 were normalized to the house-keeping gene Cyclophilin.

### Western blot

Hypothalamus and hippocampus from young (4 months of age) and aged (13 months of age) were collected and lysed with lysis buffer: 50 mM Tris, 50 mM KCL, 10 mM EDTA, 1% NP-40, supplied with protease inhibitor cocktail (Roche). Total protein tissue lysate (20 ug) from each mouse was loaded for SDS-PAGE and then detected with SRC-1 (128E7) Rabbit mAb (#2191, Cell signaling, at 1:2000) and HRP-linked anti-rabbit IgG Antibody (at 1:10000, Cell signaling).

### Luciferase assay

Neuro 2A (mouse neuroblastoma cell line) and immortalized SRC-1-KO MEF cells [17] were cultured in Dulbecco’s modified Eagle’s medium supplemented with 10% fetal bovine serum (Atlanta), 100 IU/ml penicillin and 100 ng/ml streptomycin. Luciferase reporter plasmid for S100a6 (1.66K, −1744 to −81, with primer pairs: forward 5′-AAAGGCCGTGAGAGCTAGGA-3′ and reverse 5′-TGAGGCAGTCAGTCTCAAGC-3′) and S100a11 (1.2K, −1292 to −76, with primer pairs: forward 5′-AGCTGAAATTCCAAGGGCCA-3′ and reverse 5′-TCCCCATGTCGGTGCTCTA-3′), were cloned as previous described [17].

Luciferase assay using Neuro 2A cells was performed to test if the promoters of S100a6 and S100a11 can be regulated by these transcription factors, including AP2 (gift from Robert Tjian, Addgene plasmid # 12100), Sp1 (gift from Guntram Suske, Addgene plasmid # 24543), TCF1 (gift from Kai Ge, Addgene plasmid # 40620), KLF4 (gift from Derrick Rossi, Addgene plasmid # 26815) and PU.1 (Gift from Qiang Tong) [32]. Neuro 2A cells were transfected with 800 ng of the luciferase reporter plasmid combined with 200 ng of indicated transcription factor plasmid or the control empty plasmid using the Lipofectamine LTX (Invitrogen). Cells were lysed 40 hours post-transfection, and the luciferase activity was measured using the Luciferase^®^ Reporter Assay System (Promega).

To test if SRC-1 enhances the expression of S100a6 and S100a11, SRC-1-KO MEF cells were transfected with 700 ng of the luciferase reporter plasmid combined with 200 ng of indicated transcription factor plasmid and 100 ng of pCR3.1-SRC-1 or the control empty plasmid.

## RESULTS

### SRC-1 is associated with neurodegenerative diseases

**Figure 1:**
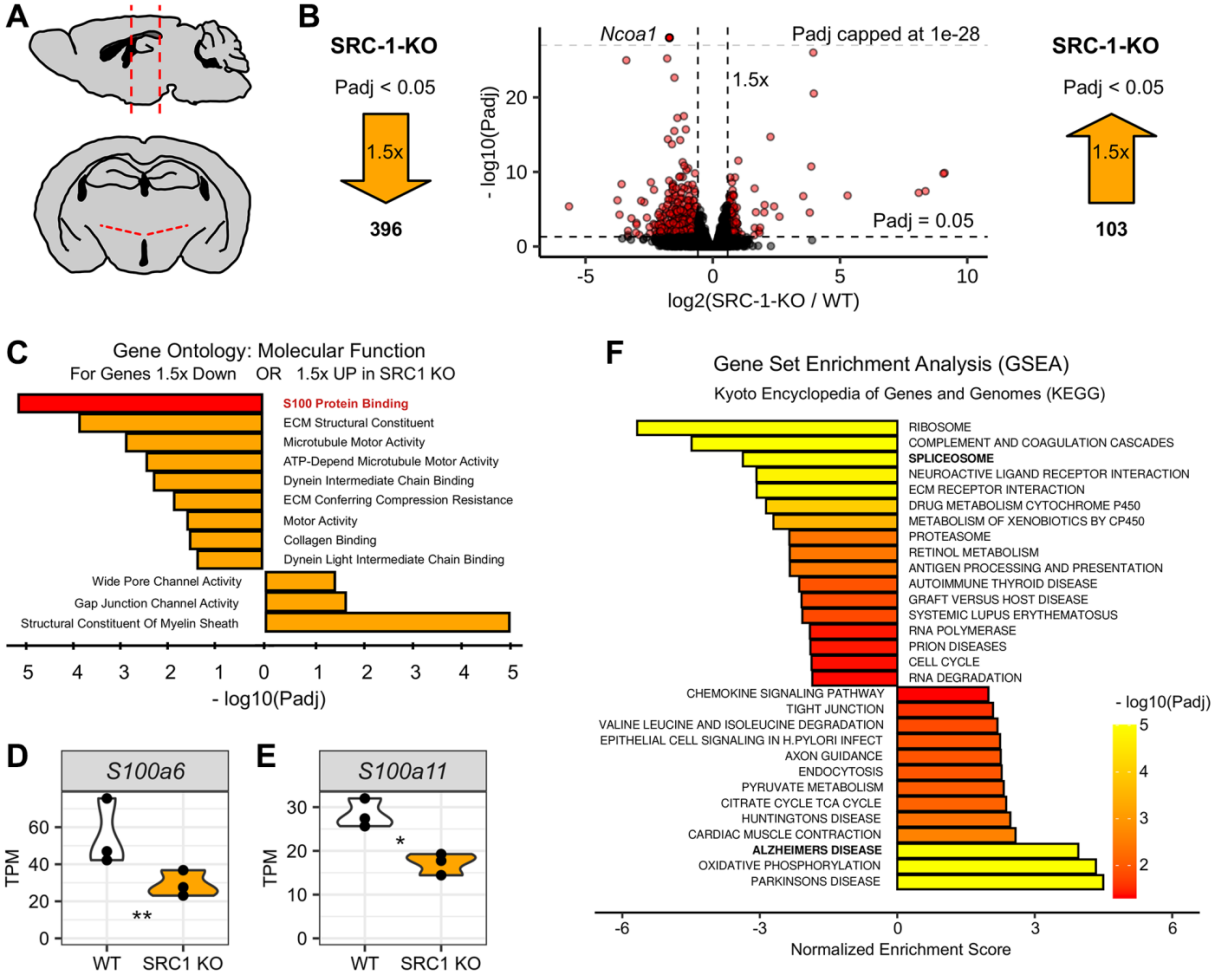
**Differential gene expression in the hypothalamus of SRC-1-KO mice.** (**A**) Diagram depicting mouse brain in sagittal view (top) and coronal view (bottom) with red dotted lines indicating hypothalamic region collected for RNA-seq experiment. (**B**) Volcano plot of RNA-seq results (center) with log_2_ fold change plotted on the x-axis and -log_10_ corrected *p*-value (*P*adj) plotted on the y-axis. Horizontal dashed line indicates *P*adj = 0.05. Two vertical lines indicate 1.5-fold either up or down. Each dot represents one gene. Genes plotted in red represent genes that are both 1.5-fold different with a *P*adj < 0.05. Light grey dashed horizontal line marks *P*adj = 1e-28 and indicates genes plotted above this line are out of the scale of the panel. Large orange arrows mark the number of genes that are 1.5-fold lower (left) or higher (right) with a *P*adj < 0.05. (**C**) Gene ontology for molecular function using genes 1.5-fold down with *P*adj < 0.05 (top) or 1.5-fold up with *P*adj < 0.05 (bottom). (**D**, **E**) Violin plots for S100a6 (**D**) and S100a11 (**D**) normalized to transcripts per million (TPM). ^*^ and ^**^, *P*adj < 0.05 or 0.01. (**F**) Gene set enrichment analysis using Kyoto encyclopedia of genes and genomes (KEGG) as output with a rank ordered gene list, using log2 fold change X -log10 *P*adj as the algorithm to rank genes, as input.

SRC-1 has been implicated to regulate both metabolic balance and cognitive functions. Importantly, the expression of SRC-1 is significantly declined with aging [22]. To explore the potential contribution of SRC-1 to aging associated diseases, including metabolic dysregulations and dementia, we performed RNA-Seq analyses using samples obtained from the hypothalamus of SRC-1-KO mice and C57BL/6 control mice (Figure 1A), and the data has been uploaded to GEO (GSE278158). As a validation, SRC-1 (coded by Ncoa1) was significantly depleted in the KO mice (Supplementary Figure 1A). Consistent with the protective role of SRC-1 in metabolic regulation, Pomc, Lepr and downstream Stat3 were significantly decreased in SRC-1-KO mice (Supplementary Figure 1B–1D). This is consistent with our previous study showing decreased leptin sensitivity in mice with deletion of SRC-1 selectively from POMC neurons, a population of hypothalamic neurons essential for metabolic regulation [17].

Applying a threshold of 1.5-fold difference and a corrected *p*-value <0.05, there were 396 genes down regulated and 103 genes up regulated in SRC-1-KO mice (Figure 1B and Supplementary data 1). We used these genes to perform Gene Set enrichment Analysis (GSEA) and Gene ontology (GO) analysis. The enrichment analysis for molecular functions revealed dysfunction in extracellular matrix binding, as well as intracellular cytoskeletal binding gene profiles. Interestingly, S100 protein binding was the most significant downregulated term, and the structure constituent of myelin sheath was the most significant upregulated term (Figure 1C). Importantly, S100 proteins and myelin functions are both implicated in the pathology of AD [33–37]. In particular, SRC-1-KO mice express significantly lower S100A6 and S100A11 (Figure 1D, 1E), and both genes have been demonstrated neuroprotective effects in neurodegenerative diseases [35, 38–47].

Further, Kyoto encyclopedia of genes and genomes (KEGG) analysis revealed that gene sets related to neurodegenerative disorders, including Alzheimer’s, were enriched, while the gene set for spliceosome was downregulated in SRC-1-KO mice (Figure 1F). Utilizing multivariate analysis of transcript splicing with an absolute inclusion level difference >0.2 and FDR <0.05, we found there were 343 significant splicing events (Supplementary Figure 1E) corroborating an altered spliceosome activity. Importantly, recent studies suggest that dysregulation of spliceosome also increases the risk of AD [48–50]. Although these splicing events are not directly involved in AD, GO analysis showed that they are involved in neuron projection organization (Supplementary Figure 1E), which is essential for normal neuron functions.

Together, the changes of gene profiles in SRC-1-KO mice suggest higher risks of neurodegenerative diseases including AD.

### SRC-1 regulates neural plasticity of the hippocampal CA1 neurons

**Figure 2:**
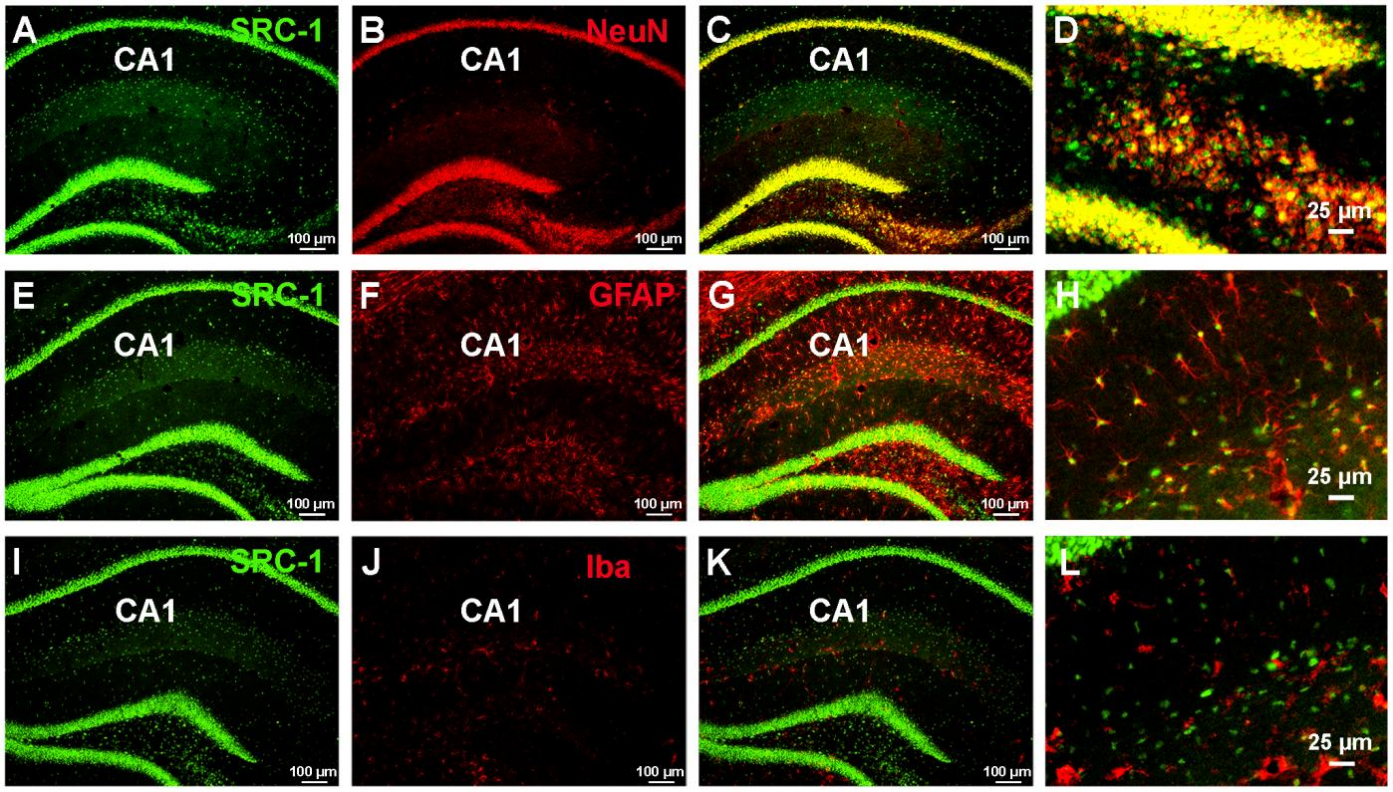
**The expression of SRC-1 in the hippocampus.** (**A**–**C**) Co-staining of SRC-1 (green, **A**) and a neuron marker (NeuN, red, **B**), and merge (**C**) in the hippocampal CA1 region. (**D**) is high magnification indicating co-expression of SRC-1 in NeuN labeled neurons. (**E**–**G**) Co-staining of SRC-1 (green, **E**) and an astrocyte marker (GFAP, red, **F**), and merge (**G**) in the hippocampal CA1 region. (**H**) is high magnification indicating co-expression of SRC-1 in GFAP labeled astrocytes. (**I**–**K**) Co-staining of SRC-1 (green, **I**) and a microglial marker (Iba, red, **J**), and merge (**K**) in the hippocampal CA1 region. (**L**) is high magnification indicating no double-labelling.

It has been reported that siRNA-mediated knockdown of SRC-1 in the hippocampal CA1 impairs memory in mice, associated with decreased CA1 synapse density, postsynaptic density thickness, and long-term potentiation (LTP) [20]. This finding led us to focus on the hippocampal CA1 region. Here we found that both neurons (marked by NeuN) and astrocytes (marked by GFAP) in the hippocampal CA1 abundantly expressed SRC-1, while microglial cells (marked by Iba) did not (Figure 2).

**Figure 3:**
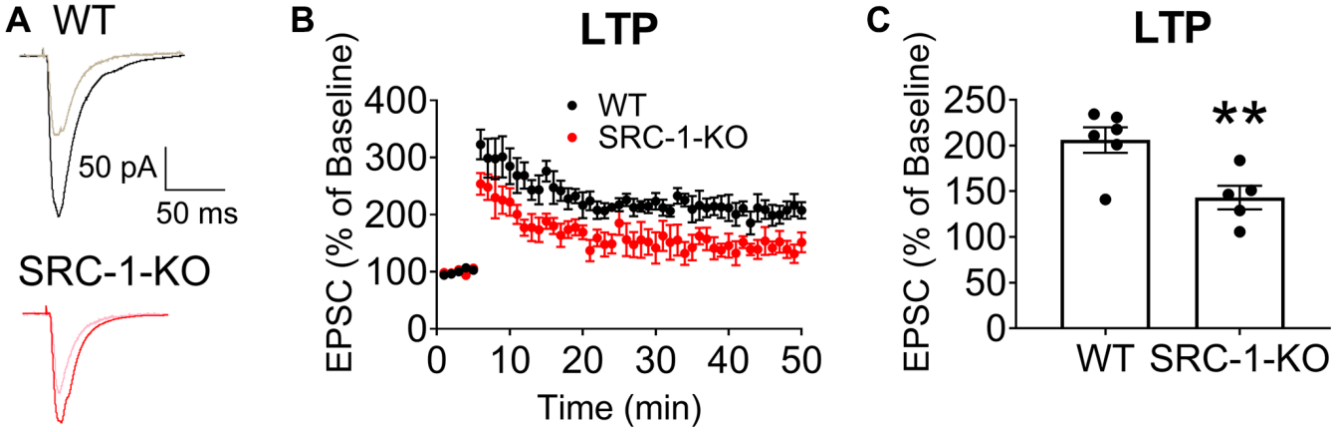
**Impaired LTP in hippocampal CA1 neurons of SRC-1-KO mice.** (**A**) Typical EPSC traces before (lighter curve) and after (darker curve) LTP induction in CA1 neurons from WT or SRC-1-KO mice. (**B**) Magnitude of EPSC elevations before (0–5 min) and after LTP induction (5–50 min). (**C**) Averaged EPSC elevations during 45–50 min in (**B**). *N* = 5-6 neurons from 3 mice. ^**^P < 0.01 in unpaired two-tailed *t*-tests.

We further explored neural plasticity in the hippocampal CA1 neurons. To this end, we used a high-frequency field stimulation (HFS) protocol to induce long-term potentiation (LTP) of evoked excitatory post-synaptic current (EPSC) in CA1 neurons from WT mice, as indicated by a sustained increase of EPSC after the HFS stimulation. However, in SRC-1-KO mice, LTP was significantly blunted (Figure 3). These results predict a protective role of hippocampal SRC-1 in cognitive functions.

### SRC-1 prevents aging-associated memory loss

**Figure 4:**
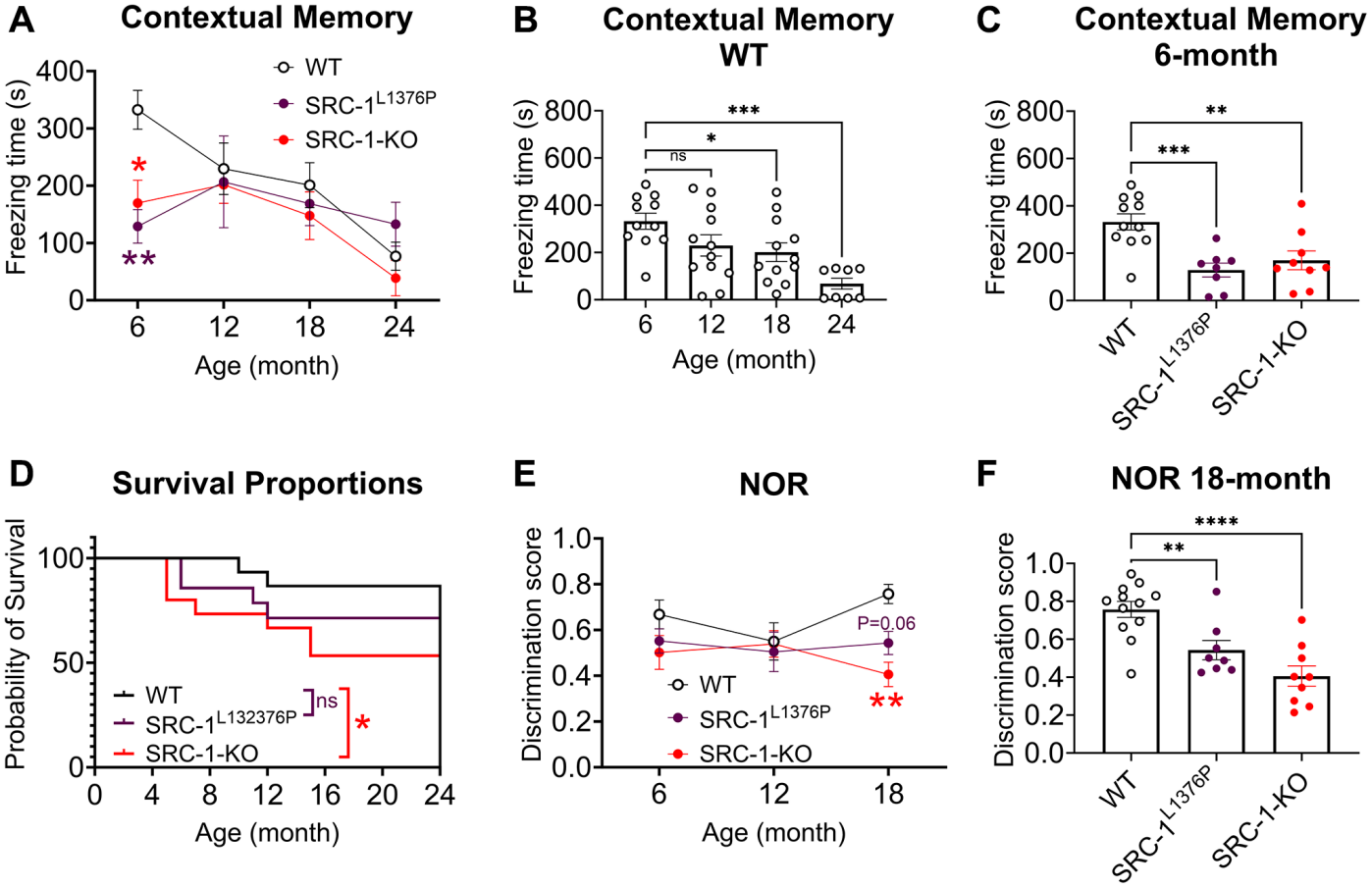
**Impaired cognitive functions of SRC-1-KO mice and SRC-1^L1376P^ mice at different ages.** (**A**–**C**) Freezing time in the fear conditioning test in all mice at different ages (**A**), in WT mice at different ages (**B**), and in all groups of mice at 6 months of age (**C**). *N* = 8–12 mice/group. Data are presented as mean ± SEM and/or with individual data points. ^*^P < 0.05; ^**^P < 0.01; ^***^P < 0.001 in in two-way ANOVA analyses followed by Tukey’s multiple comparisons test (**A**) or one way ANOVA analysis followed by Dunnett's multiple comparisons test (**B**, **C**). (**D**) The surviving curve of WT mice, SRC-1-KO mice and SRC-1^L1376P^ mice. ^*^P < 0.05 in Logrank test for trend test. (**E**, **F**) Discrimination score is defined as the ratio of time spent with the novel object during test vs. (training + test) in the novel object test in WT, SRC-1^L1376P^ and SRC-1-KO mice at different ages (**E**) and at 18 months of age (**F**). *N* = 8–12 mice/group. Data are presented as mean ± SEM with individual data points. ^**^P < 0.01; ^****^P < 0.0001 in in two-way ANOVA analyses followed by Tukey’s multiple comparisons test (**E**) or one way ANOVA analysis followed by Dunnett's multiple comparisons test (F).

To fully evaluate the function of SRC-1, we compared the cognitive behaviors among mice with either global deletion of SRC-1 (SRC-1-KO) or knock-in of a humanized loss-of-function point mutation (SRC-1^L1376P^) [17]. Similar to human, mice displayed an aging associated cognition decline. The contextual memory of WT mice was significantly decreased after 18 months of age (Figure 4A, 4B), as indicated by the decrease of freezing behavior in the contextual fear conditioning test. However, this reduction did not happen in SRC-1-KO mice and SRC-1^L1376P^ mice, because their contextual memory had already been significantly impaired as early as 6 months of age compared to WT mice (Figure 4C and Supplementary Figure 2A, 2B). Consistently, the survival rate dropped with aging in all mice, and it was significantly lower in SRC-1-KO mice and displayed a non-significant decrease trend in SRC-1^L1376P^ mice, compared to WT control mice (Figure 4D). These results together supported an early-aging phenotypes caused by SRC-1 deficiency.

In the novel object recognition (NOR) test, SRC-1-KO mice and SRC-1^L1376P^ mice displayed significant decreased discrimination score between novel object and old object at 18 months of age, indicating impaired cognition (Figure 4E, 4F). However, both SRC-1-KO mice and SRC-1^L1376P^ mice displayed similar performance to WT mice in the Radial Arm Water Maze test (RAWM), despite a non-significant trend of impaired spatial memory (Supplementary Figure 2C, 2D).

### SRC-1 expression during aging

**Figure 5:**
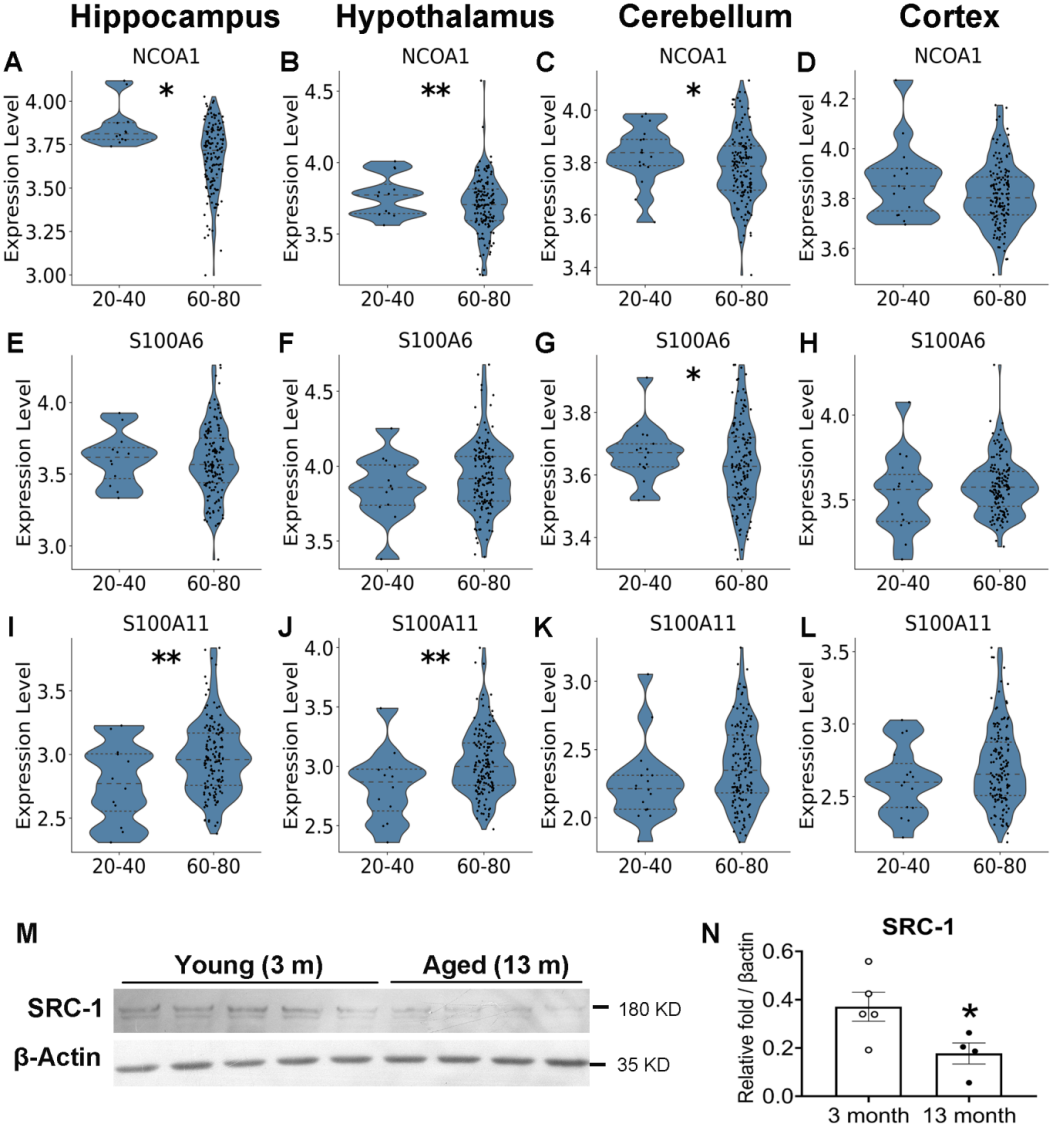
**Aging-associated changes in the expression of SRC-1, S100A6 and S100A11 in human.** Secondary analysis of published RNA-Seq data revealed the differential gene expression between young (20–40 years of age) and old (60–80 years of age) human. (**A**–**D**) The expression of SRC-1 coding gene NCOA1, (**E**–**H**) S100A6 coding gene and (**I**–**L**) S100A11 coding gene in the hippocampus, hypothalamus, cerebellum and cortex of young vs aged human brains, respectively. ^*^ and ^**^P < 0.05 or 0.01 in Wald test from DESeq2 with Benjamini-Hochberg correction for multiple comparisons. (**M**, **N**) Western Blot detection (**M**) and quantification (**N**) of SRC-1 protein levels compared to housekeeping protein β-actin in the hippocampus of young (3 months of age) and aged mice (13 months of age). *N* = 4–5 mice. ^*^P < 0.05 in unpaired two-tailed *t*-tests.

We demonstrated a protective role of SRC1 against aging associated cognitive decline, and we further explored the molecular mechanisms. We reanalyzed human public data libraries (GTEx portal) and demonstrated an age associated decline of SRC-1 expression in different brain regions, including the hippocampus, hypothalamus and cerebellum, and a non-significant decrease trend in the cortex (Figure 5A–5D). We reanalyzed published spatial RNA-Seq data to compare gene profiles between young (3 months) and old (18 months) mice brains [28]. Interestingly, we found that the expression of SRC-1 was significantly decreased in both the hypothalamus and hippocampus of aged mice compared to young mice (Supplementary Figure 3A, 3B). S100A6 displayed a non-significant decrease trend in the hippocampus and a significant decrease in the cerebellum of aged human samples, as well as a significant decrease in the hypothalamus of aged mouse samples (Figure 5E–5H and Supplementary Figure 3D, 3E). S100A11 displayed no changes or opposite changes (Figure 5I–5L and Supplementary Figure 3G, 3H). Further, secondary analysis of public bulk RNA-Seq data of mice (GSE179698) revealed a decrease trend of both SRC-1 and S100A6 but not S100A11 in the hippocampus of 18-month aged mice compared to 6-month young mice (Supplementary Figure 3C, 3F, 3I). To further confirm the age-associated decline of SRC-1 in mice, we detected the expression of SRC-1 protein using western blot. As expected, SRC-1 protein was significantly lower in the hippocampus of 13-month aged mice than that of 4-month young mice (Figure 5M, 5N).

Meanwhile, similar to reduced synaptic proteins by acute knockdown of SRC-1 in the hippocampus, the expression of Synaptophysin, GluR1 and PSD-95 [20] was also decreased in both the hippocampus and the hypothalamus of aged mice, suggesting aging associated decline of synaptic functions (Supplementary Figure 4A–4H). However, these synaptic proteins were not changed by SRC-1 deletion (Supplementary Figure 4I–4L). Based on the non-association between the expression of SRC-1 and synaptic proteins with chronic loss of SRC-1, the early-aging cognitive decline in SRC-1-KO mice should be contributed by other molecular mechanisms than these synaptic proteins.

Together, S100A6 and SRC-1 displayed synchronously decrease in both SRC-1-KO mice and aging mice, implying S100A6 as a potential downstream target gene of SRC-1.

### SRC-1 stimulates S100A6 expression

**Figure 6:**
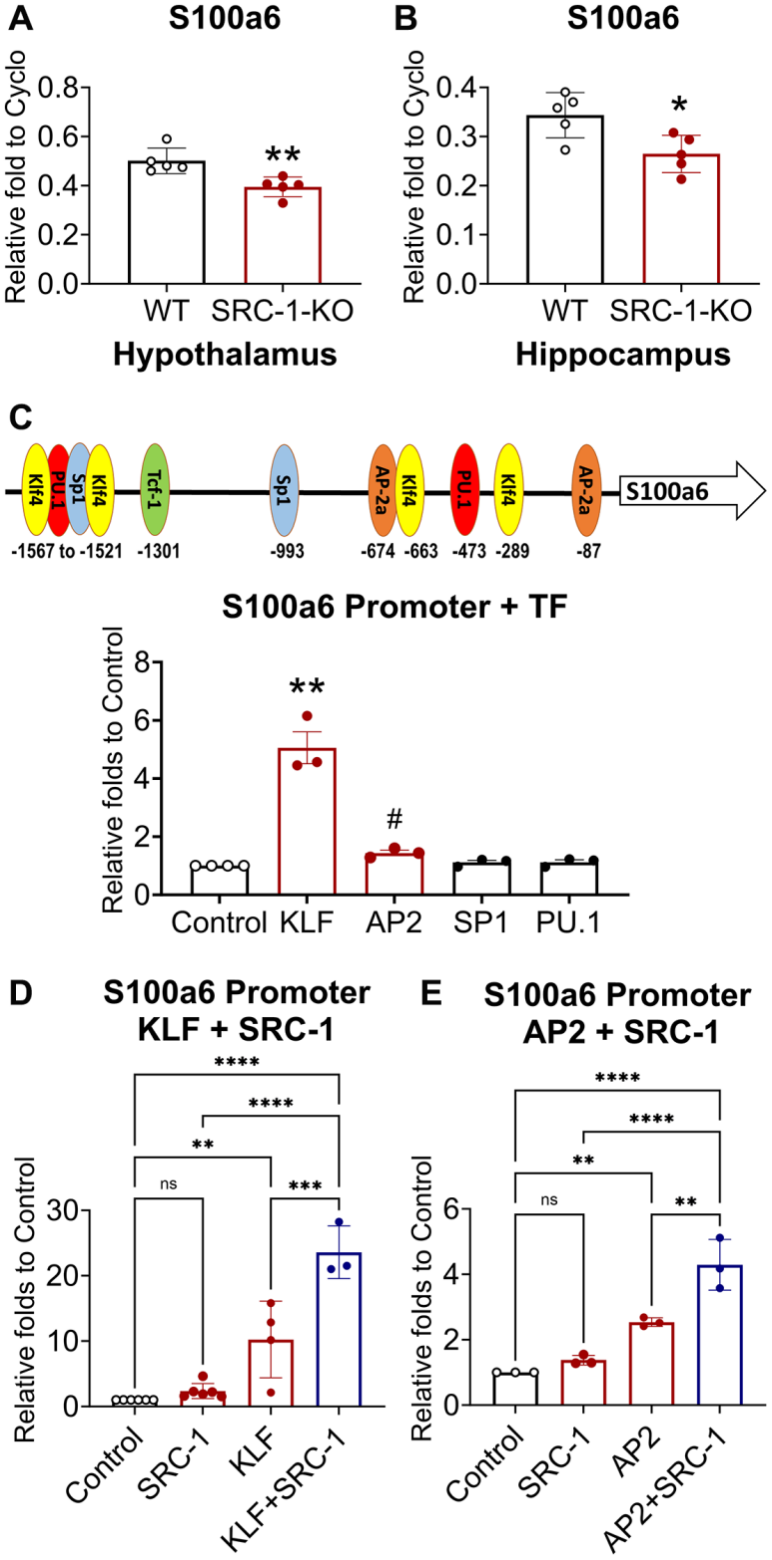
**The regulation of S100a6 by transcription factors and SRC-1.** (**A**, **B**) Relative mRNA levels of S100a6 mRNA measured in the hypothalamus (**A**) and the hippocampus (**B**) isolated from control vs. SRC-1-KO mice using Q-PCR. Data are presented as presented as mean ± SEM. *N* = 5 samples per group. ^*^P < 0.05, ^**^P < 0.01 in unpaired two-tailed *t*-tests. (**C**) Binding sites of transcription factors on the promoter of S100a6 and the effect of indicated transcription factor on S100a6 promoter luciferase activity in Neuro 2A cells. Data are presented as mean ± SEM. *N* = 3–4 repeated experiments with 3 biological replicates per group in each experiment. ^**^P < 0.001 in one-way ANOVA analyses followed by Sidak tests. ^#^*P* < 0.05 in unpaired two-tailed t-tests against Control. (**D**, **E**) Effects of SRC-1 and transcription factors KLF (**D**) and AP2 (**E**) on S100a6 promoter luciferase activity in SRC-1-KO MEF cells. Data are presented as mean ± SEM. *N* = 3–6 repeated experiments with 6 biological replicates per group in each experiment. Control group is normalized to 1 to allow comparisons among different batches of experiments. ^*^P < 0.05, ^**^P < 0.01 and ^***^P < 0.001 in two-way ANOVA analyses followed by Sidak tests.

RNA-Seq data identified that S100A6 and S100A11 were downregulated by SRC-1 deficiency in the hypothalamus. We validated the decreased expression of these two gene in the hypothalamus (Figure 6A and Supplementary Figure 5A). The hippocampus is the major center of cognition, and hippocampus CA1 neurons contribute to both NOR [51, 52] and contextual fear [53, 54]. Based on the high expression of SRC-1 in the cognition center, hippocampus CA1 neurons, we further determined whether SRC-1 regulates S100A6 and S100A11 in the hippocampus, too. S100A6 was significantly decreased (Figure 6B), and S100A11 displayed a decrease trend (Supplementary Figure 5B), in the hippocampus of SRC-1-KO mice. These results suggest S100A6 and S100A11 as two potential downstream targets of SRC-1, a co-factor for transcription factors.

To investigate how SRC-1 regulates the expression of S100A6 and S100A11, we analyzed their promoters. Using the online tool PROMO (http://alggen.lsi.upc.es/cgi-bin/promo_v3/promo/promoinit.cgi?dirDB=TF_8.3), we screened the promoter regions of S100a6 and S100a11 to identify potential transcription factors that may mediate the effects of SRC-1. These findings were further validated using JASPAR transcription factor binding profile database (https://jaspar.genereg.net/). We found several binding sites of five transcription factors, including Sp1, AP2, TCF1, KLF4 and PU.1, on S100a6 and S100a11 gene promoters (Figure 6C and Supplementary Figure 5C). These transcription factors may regulate the processes of AD [55–60]. To confirm their transcriptional activity, we used a Neuro 2A, a mouse neuroblastoma cell line, to perform luciferase assay using the promoters of S100A6 and S100A11. We found that the activity of S100A6 promoter was increased 5 folds by KLF and to a less extend by AP2, while the activity of S100A11 promoter was modestly increased by AP2 and PU.1, but not TCF (Figure 6C and Supplementary Figure 5C).

To further determine whether SRC-1 can enhance the effect of these transcription factors, we performed luciferase assays using SRC-1-KO MEF cells. As expected, both KLF and AP2 significantly increased S100A6 promoter activity. Interestingly, in the background of SRC-1 deficiency, SRC-1 supplement alone did not change S100A6 promoter activity. However, SRC-1 supplement significantly augmented the stimulatory effects of both KLF and AP2 on S100A6 promoter activity (Figure 6D, 6E). Similarly, despite that S100A11 promoter activity was not increased by SRC-1 expression, SRC-1 significantly augmented the stimulatory effects of PU.1 and AP2 on S100A11 promoter activity (Supplementary Figure 5D, 5E). Together, these results support that the co-activator activity of SRC-1. Although SRC-1 does not regulate the transcription directly, it can enhance the transcriptional activity of AP2 and KLF to promote the expression of S100A6 and enhance the transcriptional activity of PU.1 and AP2 to promote the expression of S100A11.

## DISCUSSION

The RNA-Seq analysis of SRC-1-KO brains revealed a negative correlation between SRC-1 expression and neurodegenerative diseases. Consistent with this finding, we identified a protective role of SRC-1 against aging associated cognition decline. With the decline of SRC-1 during aging [22], WT mice displayed a gradual decline of contextual memory. However, SRC-1-KO mice and SRC-1^L1376P^ mice displayed lower contextual memory throughout all the life span tested, suggesting an early-aging cognitive phenotype in SRC-1-KO mice and SRC-1^L1376P^ mice.

SRC-1-KO mice and SRC-1^L1376P^ mice displayed deficits in contextual memory as early as 6 months of age and persisted the whole life span. Consistently, contextual memory is impaired in some AD mouse models (e.g., Tg2576, 5XFAD and APP/PS1) at 4–6 months of age regardless how soon the Aβ plaques are formed [61–63]. However, 3xTg-AD and AppNL-G-F-knock-in mouse models have intact contextual memory at 6 months of age [64, 65]. Interestingly, loss of SRC-1 does not accelerate the development of Aβ plaques in APP/PS1 mice or change the expression of synaptic proteins [19]. These results argue that SRC-1 protects cognitive functions independent of Aβ pathology.

Despite acute knockdown of SRC-1 in the hippocampus reduces mice’s performance in the Morris water maze test by reducing the LTP machinery [20], our SRC-1-KO mice and SRC-1^L1376P^ mice display intact spatial memory during all the tested time in the RAWM test. In addition, while several synaptic proteins are downregulated by acute knockdown of SRC-1 in the hippocampus [20, 21], we did not detect the downregulation of these genes in SRC-1-KO mice. It is plausible that these synaptic proteins, but not the mRNAs, are dynamically regulated during memory formation. Another possibility is that the impaired contextual memory in SRC-1-KO mice and SRC-1^L1376P^ mice could be independent of the SRC-1 regulatory effects on the hippocampal synaptic proteins. It is also plausible that embryonic deletion or mutation of SRC-1 caused development changes or compensations which rescue the synaptic proteins encoding genes and/or the spatial memory deficits. Thus, SRC-1 may provide different extents of protection or play different roles in different types of memories through different mechanisms.

To explore the molecular mechanisms, RNA-Seq analysis identified S100 proteins as the most downregulated genes. S100 protein family comprised of at least 25 Ca^2+^ or Zn^2+^ binding proteins with low molecular weights. Seven S100 proteins that are present in the brain, including S100B, S100A1, S100A6, S100A7, S100A8, S100A9 and S100A12, have been implicated in regulating Aβ levels and Tau phosphorylation [35]. We identified S100A6 and S100A11 as potential downstream target genes of SRC-1, which are associated with the cognition functions and AD pathology [35, 38–47]. We also identified the transcription factors that promote the expression of S100A6 and S100A11. Further, we confirmed that SRC-1 can enhance the excitatory effects of KLF and AP2 on S100A6 promoter, and the excitatory effects of PU.1 and AP2 on S100A11 promoter, supporting the co-activator role of SRC-1. Interestingly, the combination of SRC-1 and KLF displayed the most profound stimulation on S100A6 promoter. S100A6 binds Ca^2+^ to regulate Ca^2+^ homeostasis and Ca^2+^-dependent signaling pathways, and Ca^2+^ dysregulation is implicated in AD development [40, 41]. Consistently, S100A6 is one of the most significantly positively correlated proteins with the AD phenotype [42]. S100A6 is upregulated in AD patients and in AD mouse models [43–45]. Interestingly, most S100A6 proteins are in astrocytes that surround Aβ plaques [43], and *in vitro* S100A6 treatment in mouse brain sections reduces Aβ levels and plaque burden [46]. Importantly, S100A6 expression is tightly positively correlated with cognitive function recovery in a rat model of traumatic brain injury [66], implicating the regulatory role of S100A6 in cognition. However, it is unclear whether increased S100A6 causes or defense against these phenotypes.

Previous studies indicate that acute loss of SRC-1 impairs spatial memory associated with downregulation of hippocampal synaptic proteins encoding genes. Our results support that embryonic loss of SRC-1 impairs contextual memory associated with downregulation of S100A6 and S100A11, independent of the spatial memory or hippocampal synaptic proteins encoding genes. Our studies provide SRC-1 as an upstream regulatory mechanism of S100A6. The synchronous decrease of contextual memory and the downregulation of SRC-1 and S100A6 support a protective role of SRC-1 against aging-associated memory decline, potentially through transcriptional regulation of S100A6.

Aging is associated with both metabolic dysregulations and neurodegenerative diseases, and SRC-1 contributes to both obesity and aging associated dementia. Thus, it is plausible that the decrease of SRC-1 in aging animals contributes to both aging associated body weight gain and cognition loss. Importantly, SRC-1^L1376P^ is a mutation identified from human patients with obesity, and SRC-1^L1376P^ mice are humanized mutant mouse model, which recapitulate many phenotypes observed in human patients [17]. The SRC-1 coding gene has already been added to the genetic screening panel for obesity, and our study provides a strong rationale to add this gene to the screening panel for neurodegenerative diseases, like AD.

### Limitation

Our study was conducted only on male mice to avoid the estrous cycle effects. Behavior assays were performed after 6 months of age, which corresponds to middle age in mice. The memory deficits might be developed earlier in SRC-1-KO mice and SRC-1^L1376P^ mice. We only explored the regulatory effects of SRC-1 on S100A6 and S100A11 as molecular mechanisms. Many other neurodegenerative diseases-associated molecular changes are worthwhile for future explorations. We used a promoter luciferase assay to evaluate the enhancement effect of SRC-1 on transcription factors. More comprehensive assays, like protein-protein interaction assays, need to be performed to confirm the co-activator function of SRC-1.

## CONCLUSION

We identified a negative association between SRC-1 expression and neurodegenerative diseases, and then confirmed the protective effect of SRC-1 against aging associated cognition decline. We further identified the stimulatory effect of SRC-1 on the transcription of S100A6 and S100A11, a potential molecular mechanism.

## Supplementary Materials

Supplementary Figures

Supplementary Data 1
