# Supplementary material for: A protective role of SRC-1 against aging associated cognitive decline
Source: Aging (Albany NY). 2025 Aug 27;17(8):2167–88. doi: 10.18632/aging.206311 (PMC12422791; doi:10.18632/aging.206311)
Supplement: Supplementary Figures [file aging-17-8-206311-s001.pdf]

SUPPLEMENTARY FIGURES

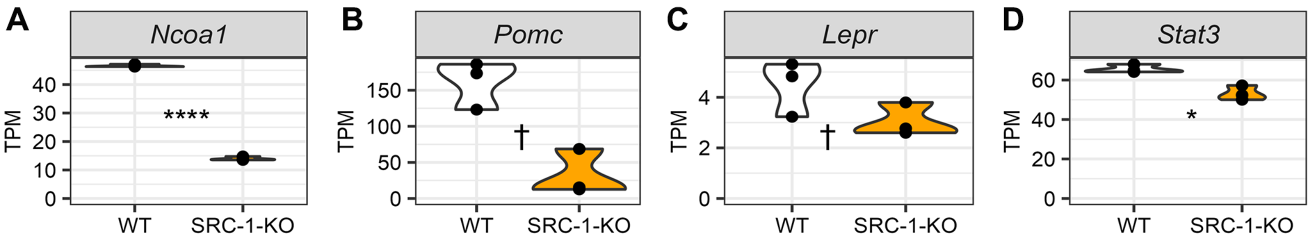

E

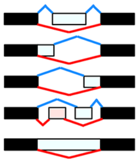

EventType	SignificantEventsJCEC	Higher in WT	Higher in SRC1 KO
SE	144	69	75
A5SS	61	29	32
A3SS	66	22	44
MXE	18	7	11
RI	54	21	33
Total	343	148	195

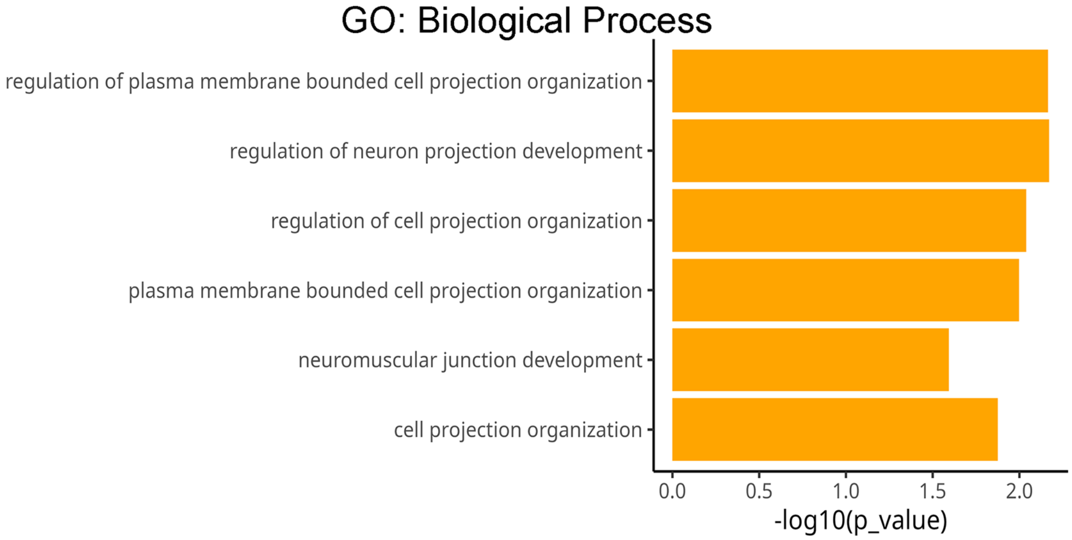

Supplementary Figure 1. Differential gene expression in the hypothalamus of SRC-1-KO mice. (**A**–**D**) Violin plots for the expression of SRC-1 coding gene Ncoa1 (**A**), POMC coding gene Pomc (**B**), leptin receptor gene Lepr (**C**) and Stat3 (**D**) based on the RNA-Seq analysis. †*P*adj < 0.1; **P*adj < 0.05; *****P*adj < 0.0001. (**E**) Multivariate analysis of transcript splicing. Table documents the total number of splicing events detected with MATS software and those deemed significant with a *P*adj < 0.05 and absolute inclusion level difference > 0.2. Splicing events were either: skipped exon (SE), alternative 5' slice site (A5SS), alternative 3' splicing site (A3SS), mutually exclusive exons (MXE), or retained intron (RI). Diagram to left of table depicts each event showing alternative pathways in red and blue. Graph below is the GO analysis for the alternative splicing events.

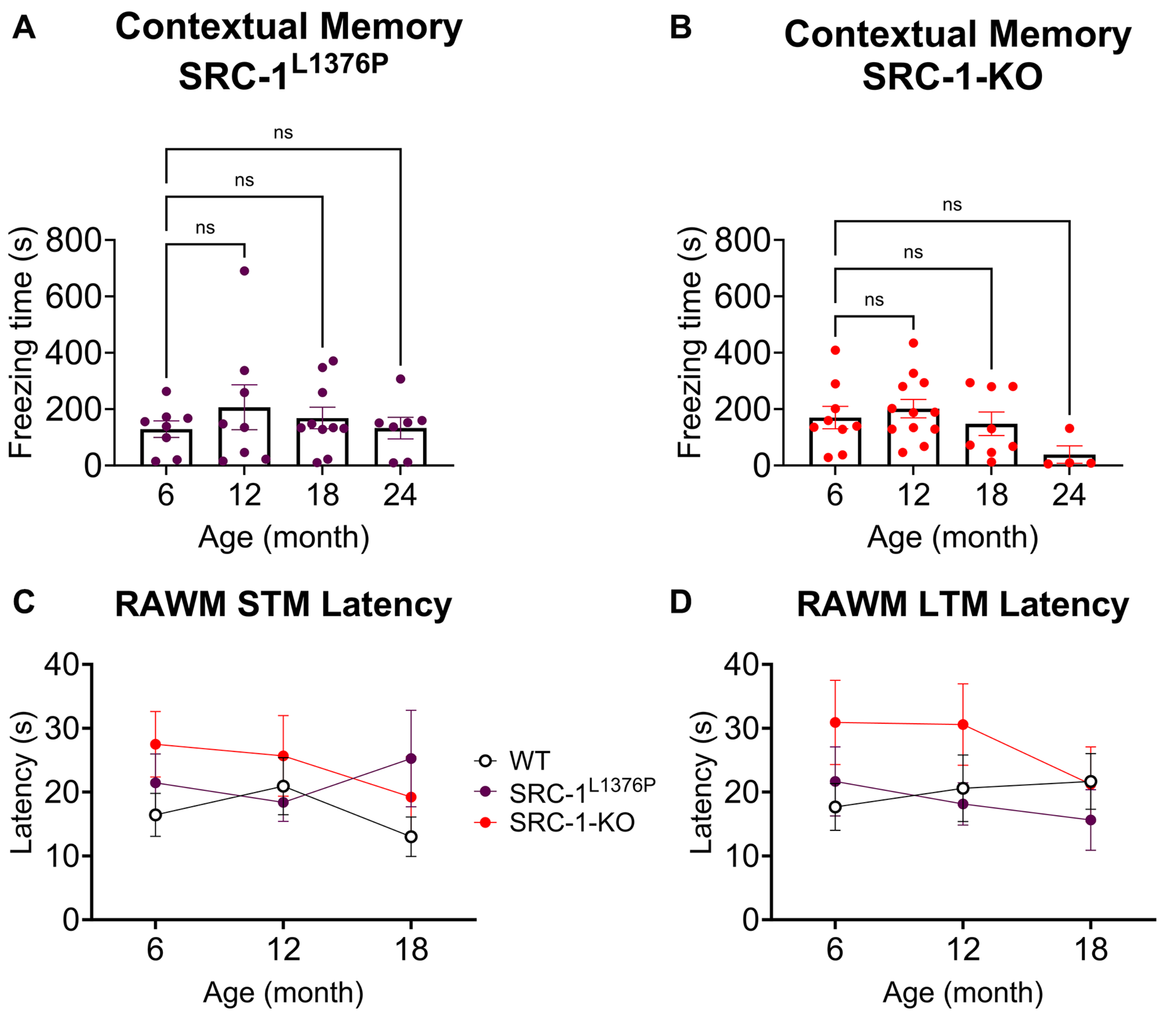

Supplementary Figure 2. Cognitive functions of SRC-1 KO mice and SRC-1^{L1376P} mice at different ages. (**A**, **B**) Freezing time in the fear conditioning test in SRC-1 KO mice (**A**) and SRC-1^{L1376P} mice (**B**) at different ages. (**C**, **D**) The latency that each mouse touched the escape platform in RAWM test at 30 minutes (**C**) or 24 hours (**D**) after the last learning session. STM, short term memory. LTM: long term memory. *N* = 8–12 mice/group. Data are presented as mean ± SEM and/or with individual data points.

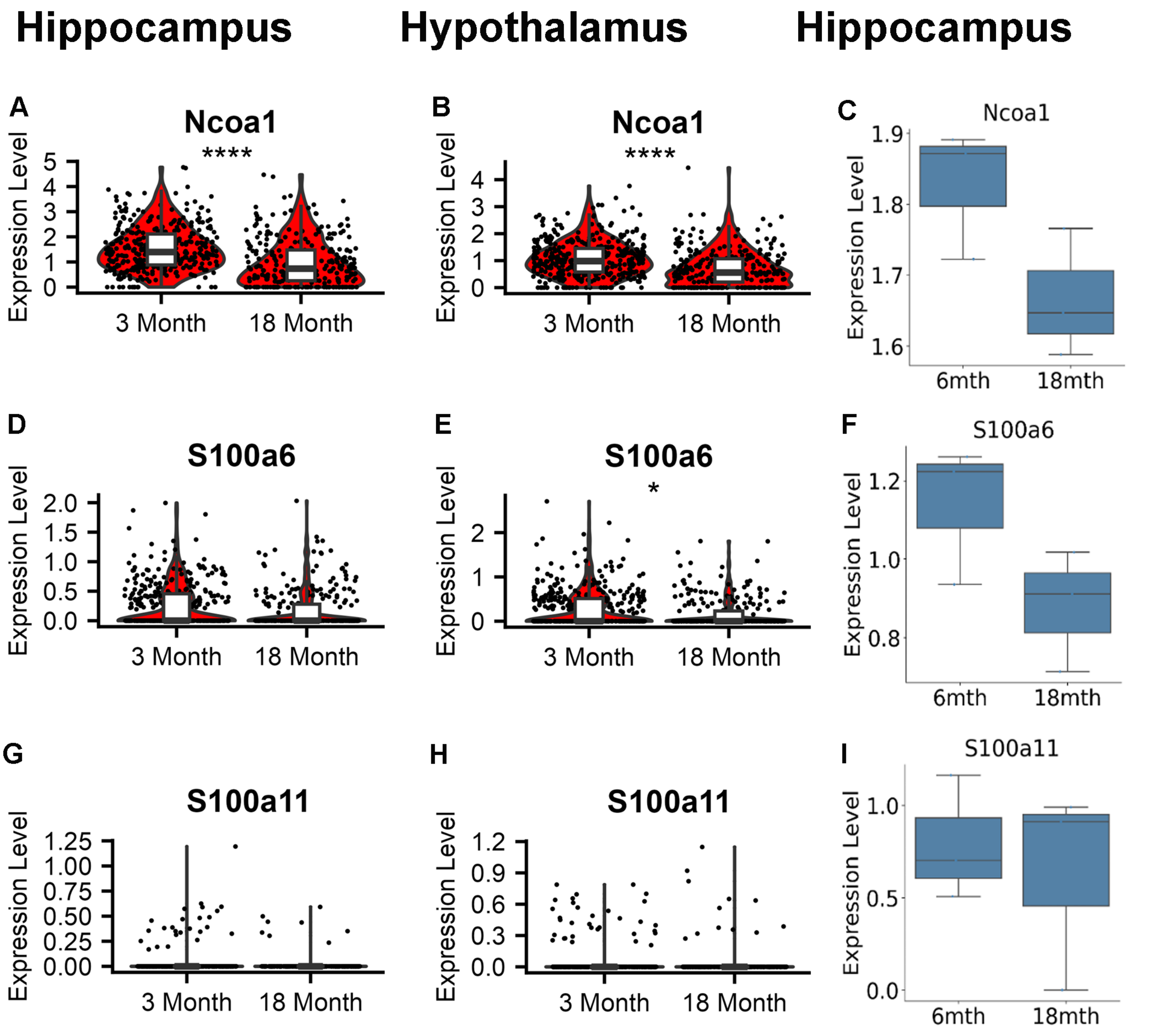

Supplementary Figure 3. Aging-associated changes in the expression of SRC-1, S100a6 and S100a11 in mice. Secondary analysis of published spatial RNA-Seq data (3 months of age vs 18 months of age) and bulk RNA-Seq data (6 months of age vs 18 months of age) revealed the differential gene expression between young and aged WT mice. (**A**–**C**) The expression of SRC-1 coding gene Ncoa1 in the hippocampus (**A**), hypothalamus (**B**), and hippocampus (**C**, bulk) of young and aged mice. (**D**–**F**) The expression of S100a6 in the hippocampus (**D**), hypothalamus (**E**), and hippocampus (**F**, bulk) of young and aged mice. (**G**–**I**) The expression of S100a11 in the hippocampus (**G**), hypothalamus (**H**), and hippocampus (**I**, bulk) of young and aged mice. * and **** $P < 0.05$ or 0.0001 in Wilcox test followed by Bonferroni correction for multiple comparisons via Seurat 5.1.0. $N = 3$ for **C**, **F** and **I**.

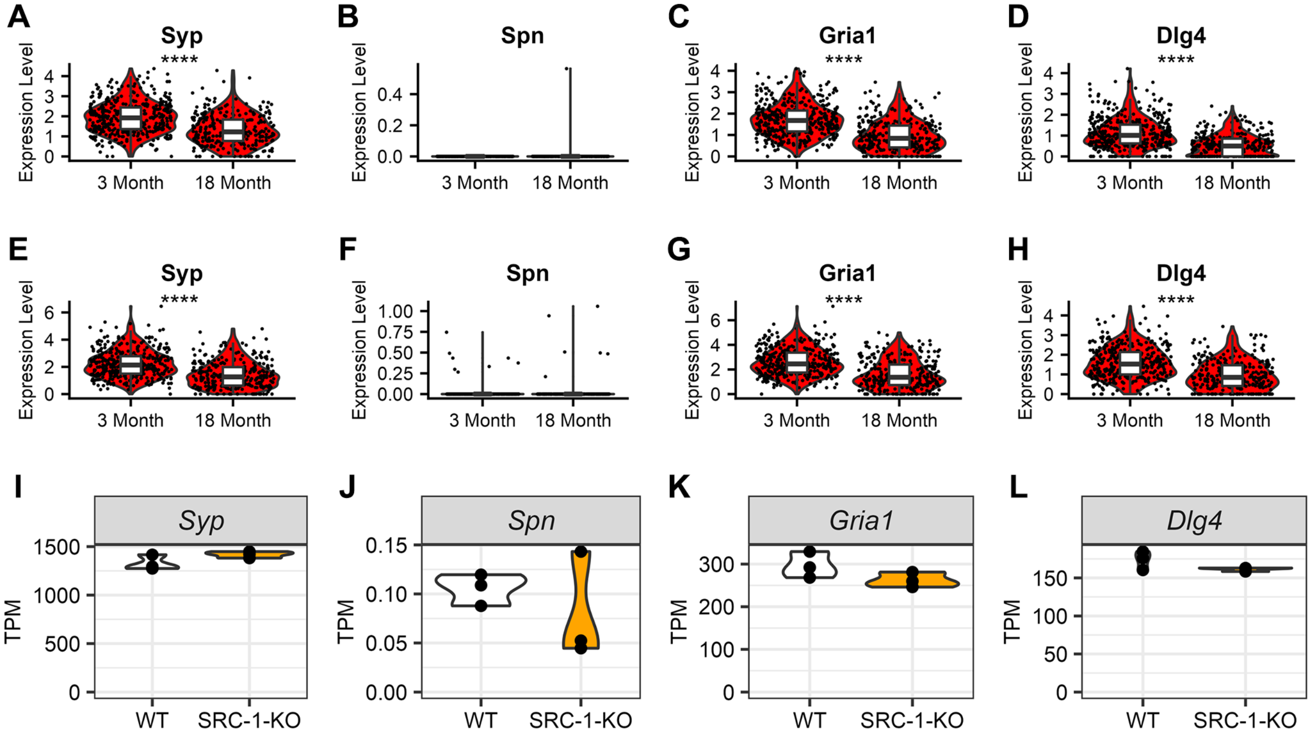

Supplementary Figure 4. The expression of synaptic proteins in aged mice and SRC-1-KO mice. (**A**–**D**) Secondary analysis of published spatial RNA-Seq data revealed the differential expression of synaptic protein coding genes, including Synaptophysin (**A** and **E**), Spinophilin (**B** and **F**), GluR1 (**C** and **G**) and PSD-95 (**D** and **H**), in the hippocampus (**A**–**D**) and hypothalamus (**E**–**H**) of young (3 months of age) and old (18 months of age) WT. (**I**–**L**) RNA-Seq analysis revealed the differential expression of synaptic protein coding genes, including Synaptophysin (**I**), Spinophilin (**J**), GluR1 (**K**) and PSD-95 (**L**), in the hypothalamus of young WT and SRC-1-KO mice. $^{****}P < 0.0001$ in Wilcox test followed by Bonferroni correction for multiple comparisons via Seurat 5.1.0.

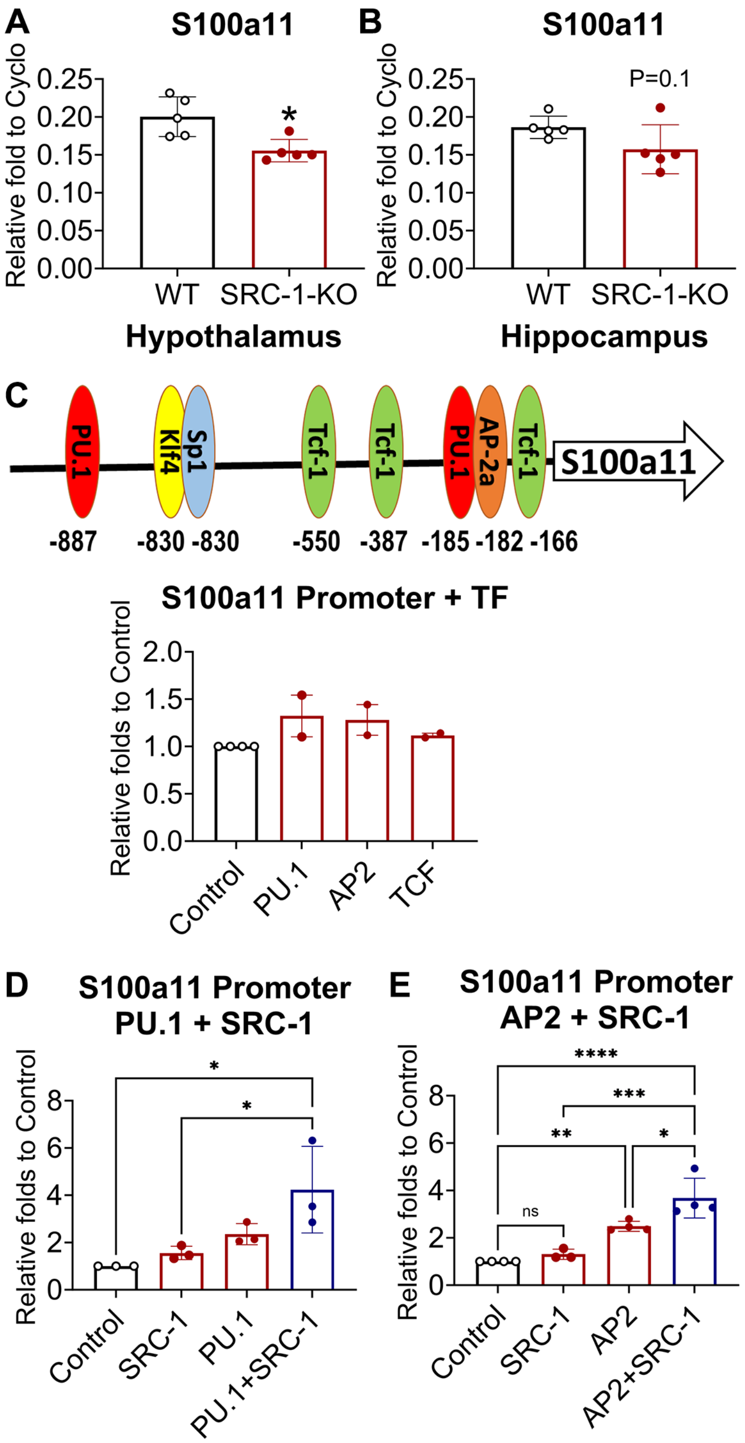

Supplementary Figure 5. The regulation of S100a11 by transcription factors and SRC-1. (**A B**) Relative mRNA levels of S100a11 mRNA measured in the hypothalamus (**A**) and the hippocampus (**B**) isolated from control vs. SRC-1KO mice using Q-PCR. Data are presented as mean ± SEM. *N* = 5 samples per group. $^{*}P < 0.05$ in unpaired two-tailed *t*-tests. (**C**) Binding sites of transcription factors on the promoter of S100a11 and the effect of indicated transcription factor on S100a11 promoter luciferase activity in Neuro 2A cells. Data are presented as mean ± SEM. *N* = 2–4 repeated experiments with 6 biological replicates per group in each experiment. (**D**, **E**) Effects of SRC-1 and transcription factors PU.1 (**D**) and AP2 (**E**) on S100a11 promoter luciferase activity in SRC-1KO MEF cells. Data are presented as mean ± SEM. *N* = 3–4 repeated experiments with 3 biological replicates per group in each experiment. Control group is normalized to 1 to allow comparisons among different batches of experiments. $^{*}P < 0.05$, $^{**}P < 0.01$ and $^{***}P < 0.001$, $^{****}P < 0.0001$ in one-way ANOVA analyses followed by Sidak tests.
